# Multiple haploids, triploids, and tetraploids found in modern-day “living fossil” *Ginkgo biloba*

**DOI:** 10.1038/s41438-018-0055-9

**Published:** 2018-10-01

**Authors:** Petr Šmarda, Lucie Horová, Ondřej Knápek, Heidi Dieck, Martin Dieck, Katarína Ražná, Pavel Hrubík, Laszlo Orlóci, Laszlo Papp, Kristýna Veselá, Pavel Veselý, Petr Bureš

**Affiliations:** 1 Department of Botany and Zoology, Masaryk University, Koltlářská 2, CZ-61137 Brno, Czech Republic; 2 Herrenkamper Gärten, Herrenkamp 1, DE-27254 Siedenburg, Germany; 3 Department of Genetics and Plant Breeding, Slovak University of Agriculture in Nitra, Tr. A. Hlinku 2, 949 76 Nitra, Slovakia; 4 Slovak University of Agriculture in Nitra, Faculty of Horticulture and Landscape Engineering, Dunajská 16, 949 11 Nitra, Slovakia; 5 Botanical Garden of Eötvös University, Illés utca 25, Budapest, Hungary

## Abstract

*Ginkgo biloba*, the last extant representative of a lineage of Mesozoic gymnosperms, is one of the few seed plants with an exceptionally long (~300 Myr) evolutionary history free of genome-wide duplications (polyploidy). Despite this genome conservatism, we have recently found a viable spontaneous tetraploid *Ginkgo* sapling during routine screening of several plants, demonstrating that natural polyploidy is possible in *Ginkgo*. Here we provide a much wider flow cytometry survey of ploidy in some European *Ginkgo* collections, and own seedlings (>2200 individuals and ~200 cultivars). We found a surprisingly high level of ploidy variation in modern-day *Ginkgo* and documented altogether 13 haploid, 3 triploid, and 10 tetraploid *Ginkgo* plants or cultivars, most of them being morphologically distinct from common diploids. Haploids frequently produced polyploid (dihaploid) buds or branches. Tetraploids showed some genome size variation. The surveyed plants provide a unique resource for future *Ginkgo* research and breeding, and they might be used to accelerate the modern diversification of this nearly extinct plant lineage.

## Genetics: Mutant Ginkgos occur with surprising frequency

The genetic abnormality, polyploidy, which underlies some unusual varieties of *Ginkgo biloba*, is more common than previously thought. Genome-wide duplications called polyploidies occur repeatedly in the evolution seed plants. The ancient Ginkgo tree, widely cultivated for its esthetic and medicinal properties, is one of the few seed plant species with a very long polyploidy-free evolutionary history. Yet, when Petr Smarda, of Masaryk University, in Brno, Czech Republic, and colleagues screened more than 2200 Ginkgo individuals and around 200 cultivars, they found 13 haploids, 3 triploids, and 10 tetraploids. These had unusual characteristics such as being shorter than normal and having especially small or large leaves. Improved understanding of the genetic mutations underlying these varieties could facilitate efforts to preserve them and help accelerate diversification of this ancient and nearly extinct plant lineage.

## Introduction

*Ginkgo biloba* L. is the last representative of Ginkgoales, a group of gymnosperms that was an important component of “temperate” forest in the Mesozoic^1,2^. Ginkgoales’s diversity has dramatically declined during the Cenozoic. Nevertheless, *Ginkgo* was still spread throughout the northern hemisphere in the Neogene^3–5^. Its distribution, however, was dramatically reduced during the Quaternary and the most recent ice ages, and only a few populations survived in isolated locations in China^5,6^. Though these locations are considered natural refugia, *Ginkgo* survival was also undoubtedly supported by its widespread planting by Buddhistic monks^5^. The phylogenetic uniqueness, the ancient nature, and the persistent morphological resemblance of *Ginkgo biloba* to its fossil relatives lead people to call it a “living fossil”.

*Ginkgo* trees were first introduced from China to Europe around the middle of the eighteenth century^5^. It was initially spread as a curiosity to botanical gardens, but its excellent prosperity in urban environments, its indisputable beauty, and its medicinal value have caused its cultivation and popularity to increase dramatically in recent decades. Today, *Ginkgo* fans are found worldwide planting this “living fossil” from sampled seeds. Gardeners have produced over 200 cultivars, most of them originating in the last three decades^4,7,8^.

This massive artificial planting and selection maintain peculiar genetic variants of *Ginkgo* that would otherwise have limited chances to establish in the wild. One of the most important mutations is polyploidy, i.e., whole-genome duplication^9–11^. Polyploidy has occurred quite frequently during the evolution of angiosperms, where it likely assisted in their massive Cenozoic diversification and their dominance of present-day vegetation^12–15^. In gymnosperms, however, polyploidy is rare^16–20^. Moreover, *Ginkgo* is among the few lineages of gymnosperms (and seed plants as a whole) with exceptionally long evolutionary histories free of whole-genome duplication^21^. The last known duplication occurred in the common ancestor of *Ginkgo* and cycads about 300 Mya^22^. Polyploid or haploid *Ginkgo* tissue cultures have already been obtained in vitro^23,24^, though plant regenerants were never successfully established, and other methods for polyploidy induction also seem ineffective in *Ginkgo*^25^. Despite this, we recently found a viable spontaneous tetraploid *Ginkgo* sapling in the Botanical Garden of Masaryk University in Brno, Czech Republic (Table 1) during routine genome size screening of some plants grown from the seeds of three females cultivated in this garden^26^. This finding demonstrated that natural polyploidy exists in *Ginkgo*, and we suggested^26^ that screening other existing *Ginkgo* collections may be an effective way of searching for other *Ginkgo* plants with unusual ploidies. These may eventually be used as mating partners for our tetraploid, for other breeding purposes, and for further experimentation.

**Table 1:** Basic characteristics of *Ginkgo* plants and cultivars with unusual ploidy levels (for details of leaves see Fig. 1)

Plant/cultivar name	Ploidy^a^	Screened collection^b^	Place of origin/producer	Mode of origin^c^	Habit/growth	Sex	Year of appearance
Anny’s dwarf	1x, 1x > 2x, 2x	BHW (1x > 2x), L (2x)	André van Nijnatten Boomkwekerij, Zundern, Neederlad	Seedling	Dwarf	JUV^d^/M^ef^	1993
Baldii	1x	HL (1x), W (1x > 2x)	? Wiel Linssen, Neederland	?	Dwarf, weeping	M^f^	?
Barabit´s fastigiata	1x, 1x > 2x	HL (1x), W (1x > 2x)	Elmér Barabits Löver Pinetum, Sopron, Hungary	Seedling	Dwarf, upright	M^f^	1980s
Clica (=Folkers selection)	1x	LWA^g^	Albert-Arboreticum, USA	?	Dwarf	?JUV	?
Fastigiata	1x, 1x > 2x, 2x	1x (N), 1x > 2x (W), 2x (HTLC)	?	?Seedling	Semidwarf, upright	M^ef^	? end of 19th century
Chase Manhatten	1x > 2x	HB	Bob Hartline, USA	?	Dwarf	M^ef^	?
Chris’ dwarf	1x, 1x > 2x	1x(BOL), 1x > 2x(W)	USA	?	Dwarf, weeping	M^ef^	?
Little Joe	1x, 1x > 2x	1x(H), 1x > 2x(WA)	Josef Hanhl tree nursery, Austria	Seedling	Dwarf	M^f^	2002–2007
Menhir	1x	1x(BL), 1x > 2x(HA)	Hazerswoude nursery, Neederland	Seedling	Semidwarf, upright	–	1996
Munkchin	1x, 1x > 2x	1x(BH), 1x > 2x(W)	? USA	?	Dwarf	F^ef^	?
Nory’s	1x	B	Francesco Vignoli Vivai, Italy	Mutation of haploid cv. Obelisk	Dwarf	JUV^d^	~2008
Obelisk	1x	BHL	Francesco Vignoli Vivai, Italy	Seedling	Semidwarf, upright	JUV^d^/M^f^	2006–2008
Rocky	1x	H	Bill Janssen, Collectors nursery, USA	?	Dwarf, upright	?JUV	<2006
Knápek’s triploid	3x	P	Tropik Hukvaldy garden shop, Czech Republic	Seedling	Normal	JUV^d^	2002
Orlóci triploid	3x	T	Laszlo Orlóci, BG Budapest, Hungary	Seedling	Normal	JUV^d^	~2005
Ožana’s triploid	3x	P	Vladimír Ožana tree nursery (Czech Republic)	Seedling	Normal	JUV^d^	2014
Blažíčková’s tetraploid	4x	P	Jana Blažíčková, Czech Republic	Seedling	?Normal	JUV^d^	2015
Bullwinkle	4x	W	Bill Janssen, Collectors nursery, USA	?	?Normal	M/F^f^	?
Freak	4x	BH	Josef Hanhl tree nursery, Austria	Seedling	?Normal	?M^f^	1993–1997
Jagged Jade	4x	W	?	Mutation of diploid cv. Jade Butterflies	?Semidwarf	?M^h^	?
Masaryk University tetraploid	4x	BG MU Brno	BG Masaryk University in Brno, Czech Republic	Seedling	Normal	JUV^d^	~2012
Pendula Gruga	4x	BHN^i^	Siegfried Holzschuher, Gruga Park in Essen, Germany	Seedling	Weeping	JUV^d^	?
Adam	4x	A	Josef Hanhl tree nursery, Austria	Seedling	Normal	JUV^d^	2002–2007
Curly leafs	4x	A	Josef Hanhl tree nursery, Austria	Seedling	Normal	JUV^d^	2002–2007
Horizontalis	4x	A(4x), H(2x)	? BG Leiden, Neederland	?Mutation	Weeping	?	?old cultivar
Hoss	4x	A	Josef Hanhl tree nursery, Austria	Seedling	Normal	JUV^d^	2002–2007

M male, *F* female, *JUV* not flowering yet

^a^ 1x—haploids, 1x > 2x—haploids forming spontaneous diploid (dihaploid) leaves, buds, or branches, 3x—triploids, 4x—tetraploids

^b^ Coding of collections: A—Josef Hahnl tree nursery (Austria), B—BG Budapets (Hungary), H—Herrenkampen Gärten (Germany), L—Ovocné a okrasné školky Litomyšl (Czech Republic), N—BG Nitra (Slovakia), O—Vladimír Ožana tree nursery (Czech Republic), P—personal collection of the grower/founder, T—Hungarian National cultivar collection, Tordas (Hungary), W—Manfred Bindewald arboretum (Germany)

^c^ Seedling = selection from spontaneous seedlings

^d^ Has not flowered yet according to the personal communication with the grower/breeder

^e^Coder (2003)^40^

^f^ Bego (2011)^4^

^g^ Both plants named originally as “Clica” and “Folkers selection” were measured

^h^ Mother cultivar is male

^i^ Grown under the name “Pendula” in N

In this paper, we describe the screening of ploidy variation in *Ginkgo* seedlings, cultivated trees, and available cultivars, including in total over 2200 *Ginkgo* individuals and ~200 *Ginkgo* cultivars. In addition to exact ploidy detection and genome size estimations using flow cytometry, we also investigated the possibility of ploidy identification by measurement of stomatal guard cells and pores.

## Material and methods

### Material

Several different sources (i–v) of *Ginkgo* plants were used for measurements of ploidy level in our study. The largest part represented (i) seedlings grown from seeds of maternal trees of the known tetraploid sapling found in the Botanical Garden of Masaryk University in Brno, Czech Republic (BG Brno). Ripened *Ginkgo* seeds fallen from all four diploid female trees were continuously collected for this experiment in autumn 2015. To remove fleshy outer seed coat, seeds were placed in a porous plastic garden crate immersed in water and mashed by hand; washed seeds were then surface-sterilized for a few minutes in a weak solution of sodium hypochlorite, rinsed in water, and maintained under room conditions for 1 day to dry their surfaces. The cleaned seeds were placed into wet vermiculite and stored in plastic zip-bags in a dark refrigerator at ~4 °C until seeding time. Some seeds were also kept to ferment and were stored in natural outside conditions over the winter (experiencing multiple frost periods). Fleshy outer seed coat was removed from these seeds in the same way several days before the seeding. Altogether, 3570 seeds were sown at the beginning of April 2016 in seedling trays (45 ml per chamber) in a sand:chernozem:garden–compost substrate mixed at a ratio of 1:2:2. Seedling trays were placed in the outside experimental garden and occasionally watered. Of the 3570 seeded *Ginkgo* seeds, 1360 emerged, producing in total 1533 individual seedlings. The extra seedlings came from 163 seeds that produced two seedlings and 5 seeds that produced three; i.e., 168 (12.8%) emerging seeds were polyembryonic. Seeds stored in the refrigerator before sowing showed slightly higher emergence (42.5%) than naturally stored seeds (35%). Throughout 2016, leaves from all seedlings that emerged were collected for flow cytometry measurements. No new seedlings emerged in 2017.

Further, ploidy was determined also (ii) in seedlings and young saplings produced from seeds of local *Ginkgo* trees by Czech *Ginkgo* fans and growers (Table S1), (iii) in various other trees sampled opportunistically by ourselves or sent to us for analysis by various *Ginkgo* fans (Table S1), and (iv) in deep-frozen leaves from 79 *Ginkgo* trees cultivated across Slovakia which were sampled for other genetic analyses^27^ (Table S2). Ploidy screening also included (v) various *Ginkgo* cultivar collections and assortment of local *Ginkgo* sellers (Table S3). Altogether, 371 plants of about 200 cultivars were analyzed for ploidy level in our survey (Table S3).

### Ploidy and genome size

Measurements of ploidy level and genome size were done using flow cytometry. The ploidy measurements were conducted using a Cy Flow ML (Partec, Germany) flow cytometer, AT-specific 4′,6-diamidino-2-phenylindole (DAPI), and an external standard. To test for possible aneuploidy or intraploidy differences in genome size, the DAPI measurements were repeated with fresh green leaves from plants with unusual ploidies using additionally the internal standards *Pisum sativum*^28^ or *Vicia faba*^28^ (2C = 7841 and 23,273 Mbp, respectively^29^). These measurements provided information about the relative genome size (Table S4). In selected plants, we also measured directly the absolute genome size (somatic nuclear DNA content) using the same internal standards and intercalating propidium iodide (PI) dye.

The preparation of samples followed Otto^30^ and was similar for all flow cytometry measurements. A piece of leaf was chopped using a sharp razor blade (in the case of internal standardization, it was chopped together with the leaf of the standard) in a Petri dish containing 0.5 ml of Otto I buffer (0.1 M citric acid, 0.5% Tween 20). To improve the signal/background ratio, the original Otto I solution was mixed 1:1 with 0.1 M hydrochloric acid and supplied with additional Tween 20 (4.4% final concentration). An additional 0.5 ml of “improved” Otto I buffer was added. The crude nuclear suspension was filtered through 50-µm nylon mesh. The filtered suspension was finally supplemented with 1 ml of Otto II buffer (0.4 M Na_2_HPO_4_·12H_2_O) supplemented with either DAPI (final concentration 2.0 µg/ml) or PI (final concentration 50 µg/ml), depending on the type of the measurement. Using DAPI dye and the above procedure, we obtained DNA signal and were able to detect ploidy even in (i) yellow, fallen; (ii) 1-year-old, deep-frozen; and (iii) few-days-old, dried *Ginkgo* leaves. At least 1000 nuclei were analyzed for measurements of ploidy level and at least 5000 nuclei for measurements of relative or absolute genome size. Measurements of absolute genome size were repeated three times on different days per each sample and finally averaged.

### Stomatal parameters

**Fig. 1:**
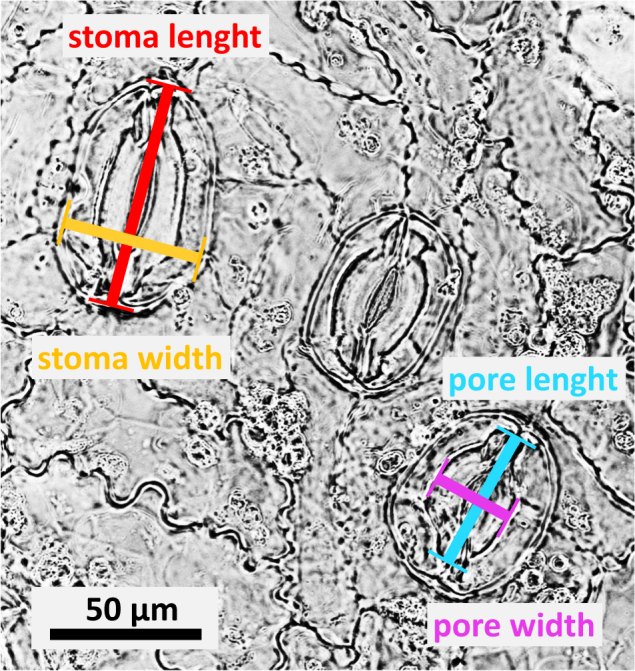
Epidermal peel of abaxial leaf side of *Ginkgo biloba* (Knápek´s triploid, sample nr. 40) showing the measured stomatal parameters (Tab. S5). Stoma length—length of the sunken guard cells measured in longitudinal stoma axis; stoma width—measured as the largest distance found between outer guard cell walls perpendicularly to the longitudinal stoma axis; pore length—size of the pore (visible on leaf surface) measured in longitudinal stoma axis; pore width—the largest size of the pore found perpendicularly to the longitudinal stoma axis

In addition to the exact flow cytometry measurements of ploidy and genome size, we further attempt to test alternative, less instrumental-dependent methods of ploidy detection. The easiest method perhaps is measurement of stomata, particularly their guard cells^31,32^. Because stomata are partly sunken in *Ginkgo* leaves, we observed their parameters (guard cell length, stomata width, stomata area, stomatal pore length, width, and area; Fig. 1) directly in epidermal peels. Peels were prepared by boiling about 1 cm^2^ of the central part of a fresh leaf in about 25 ml of commercial household bleach (SAVO, Czech Republic; containing 1–5% sodium hypochlorite and 0.5–2% sodium hydroxide) for about 10 min, until the leaf became nearly transparent and both leaf sides were separated or nearly so. The bleached epidermal peels were washed in distilled water and mounted in glycerol. The peel of the abaxial, stomata-bearing leaf side was observed with a microscope (Olympus BX51) under ×200 magnification. For each *Ginkgo* sample, measurements were done in several leaves (usually up to five) and for at least 50 stomata in total (Table S5).

## Results

**Fig. 2:**
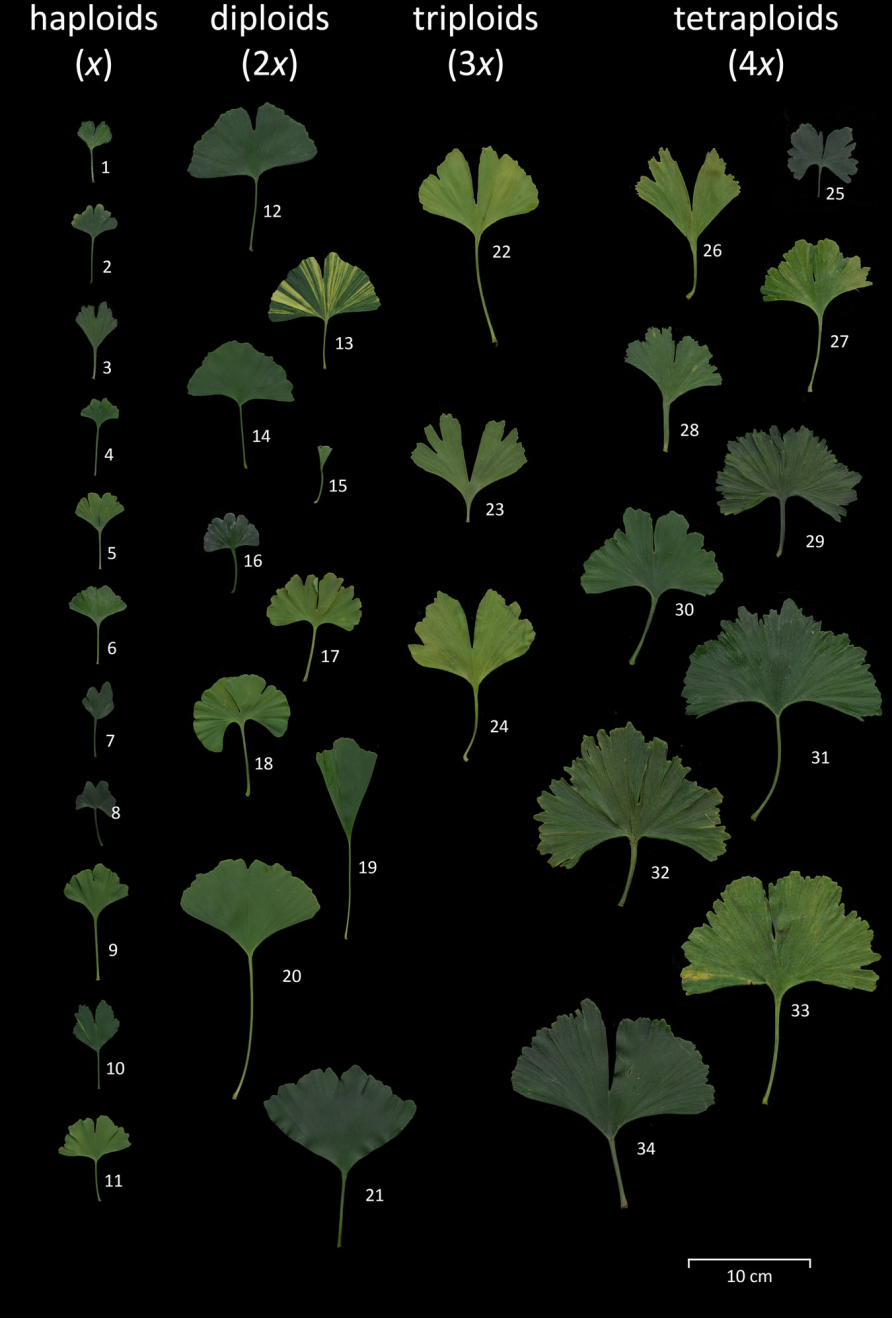
Scanned mature leaves of some selected diploid and all detected haploid and polyploid *Ginkgo* plants and cultivars. Of particular interest are the smaller leaf size of haploids, the deep central groove (lobbing) in the leaves of triploids, and the large size and finely irregularly lobed/jagged (laciniate) leaf margins in tetraploids. Scale bar = 10 cm. Haploids: 1—Clica, 2—Rocky, 3—Fastigiata, 4—Baldii, 5—Little Joe, 6—Nory´s, 7—Chris’s Dwarf, 8—Munchkin, 9—Obelisk, 10—Menhir, 11—Anny´s Dwarf. Diploids: 12—old tree BG Budapest, 13—Finger Variegated, 14—San José Gold, 15—Tubifolia, 16—Pine Glen Dwarf, 17—Mephisto, 18—Blenheim Arboretum, 19—Saratoga, 20—King of Donting, 21—Nelleke. Triploids: 22—Orlóci Triploid, 23—Ožana´s Triploid, 24—Knápek´s Triploid. Tetraploids: 25—Blažíčková´s Tetraploid, 26—Masaryk University Tetraploid, 27—Freak, 28—Bullwinkle, 29—Jagged Jade, 30—Hoss, 31—Pendula Gruga, 32—Curlie Leafs, 33—Adam, 34—Horizontalis

**Fig. 3:**
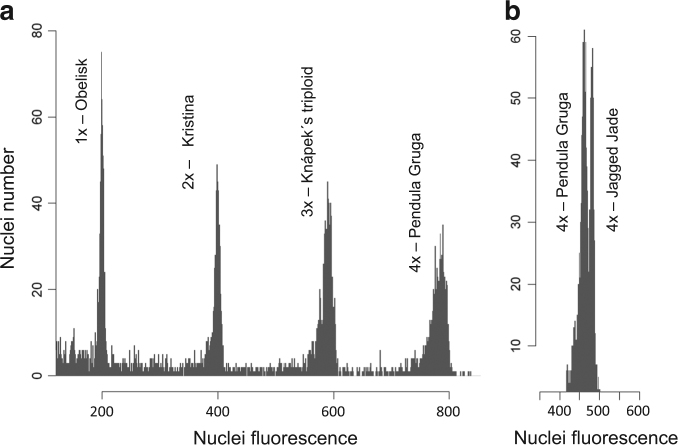
Flow cytometry histograms showing difference in relative genome size among measured Ginkgo plants. **a** among plants of all detected ploidy levels, **b** between the two most genome-size-extreme tetraploid *Ginkgo* cultivars (peak ratio = 1.041)

**Fig. 4:**
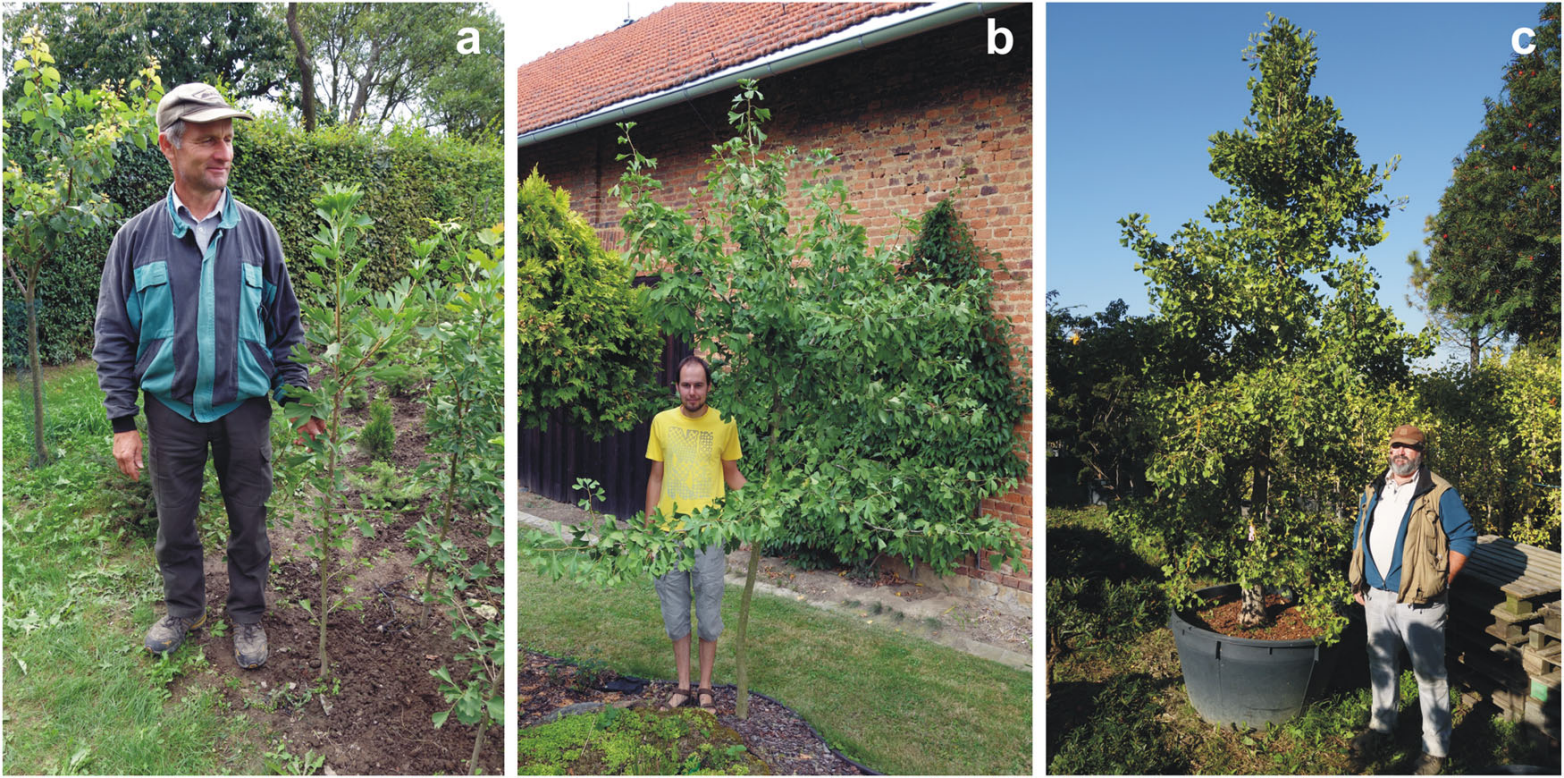
Examples of polyploid *Ginkgo* plants and their growers. **a** Vladimír Ožana with his 3-year-old triploid sapling, **b** Ondřej Knápek with his 15-year-old, pruned triploid tree, **c** Josef Hahnl with the potted, ~22-year-old mother plant of his tetraploid cv. Freak

The screening in seedling/sapling collections revealed altogether two polyploid plants: a tetraploid seedling in the collection of Jana Blažíčková and a triploid sapling in the collection of Vladimír Ožana (Table 1, S1; Figs. 2 and 4). A 15-year-old spontaneous triploid tree was found in the personal garden of Ondřej Knápek (Fig. 4) and another ~12-year-old spontaneous triploid (used as a rootstock) in a *Ginkgo* collection in Tordas, Hungary (Table 1, S1). The screening of various *Ginkgo* cultivar collections showed even much extensive ploidy variation. Of about 200 screened *Ginkgo* cultivars (Table S3), 13 were found to be haploid (Menhir, Obelisk, Fastigiata, Anny’s Dwarf, Baldii, Chris’ Dwarf, Clica, Munchkin, Barabit´s Fastigiata, Chase Manhattan, Nory’s, Rocky, Little Joe) and eight tetraploid (Adam, Bullwinkle, Curly Leafs, Freak, Horizontalis, Hoss, Jagged Jade, Pendula Gruga) (Table 1, Figs. 2, 3a and 4). In the cases of cultivars Anny’s Dwarf, Fastigiata, and Horizontalis, diploid individuals were also found (Table 1, S3).

Haploids or triploids showed no significant intraploidy genome size variation (Table S4). In tetraploids, however, genome size varied to some extent, and we observed a 1.04-fold difference between the cultivars with the largest contrast, Pendula Gruga and Jagged Jade (Table S4, Fig. 3b). The absolute genome sizes (somatic nuclear DNA contents) for selected plants/cultivars were as follows: “Obelisk” (2C = 1x = 10.16 ± 0.13 Gbp), “Kristine” (2C = 2x *=* 19.53 ± 0.48 Gbp), “Knapek´s Triploid” (2C = 3x *=* 29.19 ± 0.30 Gbp), “Pendula Gruga” (2C = 4*x* = 38.12 ± 0.71 Gbp), “Bullwinkle” (2C = 4*x* = 38.71 ± 0.56 Gbp), and “Jagged Jade” (2C = 4*x* *=* 39.41 ± 0.55 Gbp).

Ginkgo haploids seem to be characterized by smaller leaves and dwarf or upright columnar growth (Fig. 2, Table 1), though not all small-leaved and/or dwarf or upright-growing cultivars examined were haploid. All three triploids were growing well and distinguished by relatively large and deeply bilobed leaves, which in diploids are usually found only in annual shoots. Tetraploids had, as their most distinguishing characteristics, a laciniate (irregularly jagged to finely irregularly multi-lobed) leaf margin (Fig. 2). Also, their leaves were large (with the exception of a young Blažíčková´s tetraploid and Freak), robust, and thick, and they often had wide and flattened petioles (Fig. 2).

**Fig. 5:**
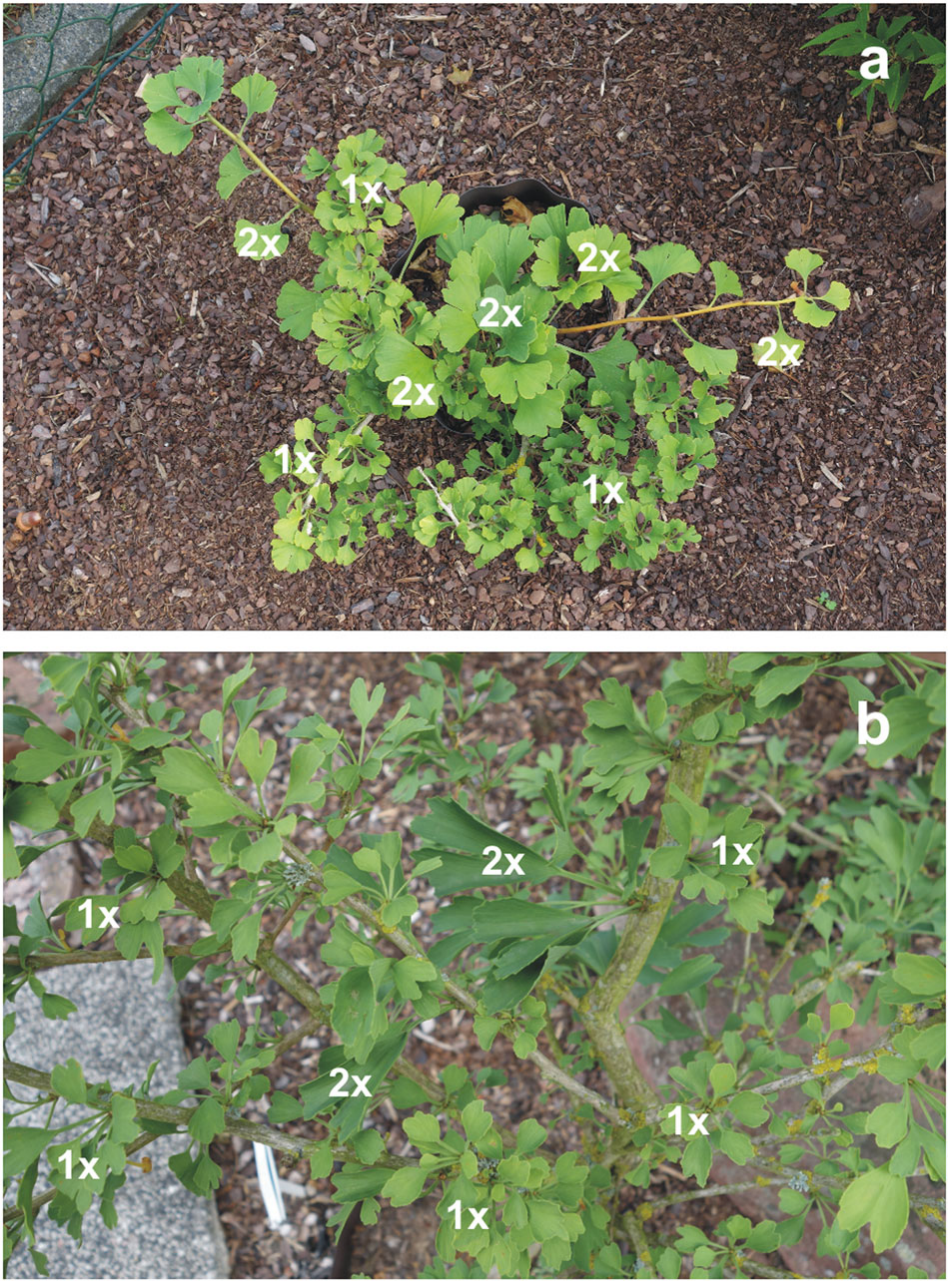
Spontaneous polyploid (dihaploid) leaf buds or whole branches (2x) are frequently formed on haploid (1x) *Ginkgo* plants. **a** cv. Chase Manhattan, **b** cv. Anny´s Dwarf; both from the personal *Ginkgo* garden of Manfred Bindewald (Germany)

In nine haploid cultivars (Anny´s Dwarf, Baldii, Barabit´s Fastigiata, Fastigiata, Chase Manhattan, Chris Dwarf, Little Joe, Menhir, Munchkin), we observed buds or whole isolated branches with apparently enlarged leaves (Fig. 5, Table 1, S3). Larger haploid plants often had several such buds or branches (Fig. 5). These enlarged leaves were usually diploid or sometimes formed with a mixture of haploid and diploid tissue (in Anny´s Dwarf, Barabit´s Fastigiata, Fastigiata, Little Joe). This indicates somatic mutation from haploid to diploid (i.e., dihaploidization) happens frequently in haploid cultivars.

**Fig. 6:**
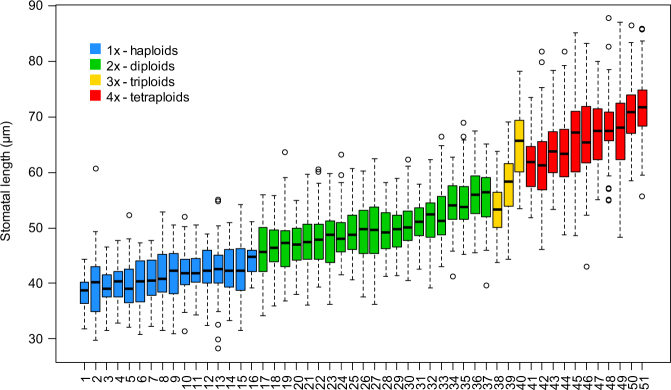
Stomatal (guard cell) lengths of different *Ginkgo* samples (cultivars) showing positive correlation with ploidy level and documenting the possibility of using this technique to detect haploid and tetraploid *Ginkgo* plants. Boxplots show: median (thick horizontal line), interquartile range (box), non-outlying values range (whiskers), and outliers (circles). Haploid samples 10, 12–16 show statistical similarity with one or more diploid samples (Tukey HSD test, *p* > 0.05); diploids always statistically differ from tetraploids; triploids cannot be distinguished in general from either diploids or tetraploids. Sample coding follows Table S5. For figures of other stomatal parameters see Figs. S1–S6

Average stomatal guard cell lengths of haploids (38.4–44.5 µm) and tetraploids (61.4–71.6 µm) were always shorter or longer, respectively, than the average among diploids (45.9–55.7 µm) (Fig. 6, Table S5). For haploids with largest stomata (Obelisk, Clica, and two samples of Chris Dwarf), however, guard cell lengths were not statistically different (Tukey HSD test, *p* > 0.05) from some small-leaved diploids (Maribo, Troll). Average guard cell lengths of triploids (53.3–64.9 µm) were intermediary between those of diploids and tetraploids and not clearly distinguishable from either of them (*p* > 0.05; Fig. 6, Table S5). A similar pattern was observed also for measurements of stomatal width and stomatal area, thought their values overlap slightly more between the different ploidies than in the case of guard cell length (Fig. S1 and S2). Pore parameters were highly correlated with the respective parameters of stomata (Adjusted *R*^2^ from linear regression of sample medians = 0.855, 0.840, 0.895 for length, width, and area, respectively; Fig. S3). However, the pore parameters frequently overlaps between different ploidies, particularly between haploids and dwarf *Ginkgo* diploids, and in general showed worse discrimination of haploid and polyploid *Ginkgo* plants (Figs. S4–S6) as compared to the guard cell length.

## Discussion

Despite the exceptional polyploidy-free evolutionary history of *Ginkgo*^21,22^, our study clearly demonstrates *Ginkgo* is capable of spontaneous polyploidy and shows that the modern European or American cultivar assortment already includes multiple *Ginkgo* plants of unusual ploidies, including several haploids, triploids, and tetraploids. The majority of such plants are relatively young, having originated in the last 20–30 years as a consequence of increased interest in *Ginkgo* and selection of plants with horticulturally attractive foliage or growth form. They are perhaps all of spontaneous origin. At least, we have no direct evidence (Table 1) that any originated by some more advanced breeding or polyploidy-inducing methods. The sowings of >10,000 seeds and consequent selection of morphologically attractive seedlings by Josef Hahnl in Austria in 1993–1997 and 2002–2007 yielded in total four tetraploid and one haploid cultivar. Together with the evidence from the other screened sowings, the frequency of appearance of unusual ploidies in semi-natural, cultivation conditions may be one in every few thousand. It is therefore likely that other spontaneous *Ginkgo* plants with unusual ploidies could be found by detailed screening of other *Ginkgo* nurseries or collections.

*Ginkgo* has naturally higher genetic diversity and a much longer tradition of cultivation in China and other Asian countries^33^, where it is mostly breed for fruit shape and seed production^7,34^. In Europe and America, however, the *Ginkgo* is cultivated and bred fast exclusively for decorative purposes, and fruiting plants are generally unwanted. The Asian *Ginkgo* collections may therefore provide different genetic types of haploid or polyploid *Ginkgo* plants, and ploidy screening in these *Ginkgo* collections is therefore worthwhile.

### Haploids

Spontaneous haploids are known in many ordinarily diploid plant species, though in gymnosperms such haploids seem quite rare^35–37^. These haploids mostly originate from a failure to fertilize an egg cell, which then continues in development as a haploid embryo^36^. Because *Ginkgo* females are heterogametic^38,39^ (ZW), this “gynogenic” pathway of haploid formation should produce an equal portion of male (Z) and female (W) haploids. However, available evidence indicates that the male sex prevails in haploids^4,40^ with the female sex reported only for cv. Munchkin (Table 1). Nevertheless, the notes of sex in *Ginkgo* haploids need to be taken with care, because haploids are usually sterile due to problems with meiosis^41^. Noting that (i) fertility of haploids is commonly restored by polyploidization^42^ (dihaploidization) and (ii) our observations of frequent formation of dihaploid buds or branches in haploids, it is not unlikely that some notes about formation of sexual organs in haploids may be related to their dihaploidized parts. Whereas dihaploidized male haploids would have the standard male chromosomal constitution (ZZ) and therefore should not have any problems with sex expression, the dihaploidy in haploid females will lead to the formation of curious superfemales (WW) whose viability remains uncertain. Whether this fact could be related to the prevalence of males in haploids remains an open question.

Haploidy in *Ginkgo* seems related to either dwarf bushy or upright columnar growth. The height of dwarf haploids usually does not exceed 3 m. The upright-type haploids grow generally more vigorously, and their height is expected to reach 5 m in cv. Menhir (US patent Nr. US PP24,226 P2). Even larger trees, up to 20 m height, are reported for haploid cv. Fastigiata^8^, a growth form already known at the end of nineteenth century^7^. However, the name “fastigiata” is used quite freely to name upright growing trees of various origins, and reports of “fastigiata” trees may also relate to diploids (Table 1, S3). The two haploid trees of cv. Fastigiata we observed both reached 4.7 m (a 16-year-old tree in BG Nitra and a 9-year-old tree in the garden of M. Bindewald). It is to be noted, however, that available growth data in *Ginkgo* haploids and polyploids (Table 1) mostly originate from grafted plants, whose growth may also depend on the vitality of the used rootstock.

Dwarfism is a common feature of plant haploids^37,43^. These dwarf plants are naturally popular with home gardeners, and preferential selection of dwarf phenotypes in *Ginkgo* nurseries is with no doubt a reason for the relatively high frequency of haploids in the modern *Ginkgo* assortment. The high frequency of *Ginkgo* haploids might also relate to the frequent polyembryony of *Ginkgo* seeds^44^, reaching 12.8% in our sowing experiment (see Materials and methods). Polyembryonic seeds show an increased rate of haploid formation in many angiosperm species and were proved as a useful source of haploids also in some conifers^37,45^. At the moment, we lack information about whether the known *Ginkgo* haploids have originated from regular or polyembryonic seeds. Nevertheless, careful inspection of twin or triplet seedlings originating from polyembryonic seeds may be one of the promising ways of searching further *Ginkgo* haploids.

Dihaploid plants will lose a number of the characters of the haploid plants (such as dwarf growth and small leaf size, Fig. 5). Therefore, dihaploid branches are removed from dwarf *Ginkgo* haploids for esthetical reasons. This is paradoxical, because such dihaploidized parts may be especially useful in further *Ginkgo* breeding, considering that the production of dihaploids is a frequent target of modern, advanced crop breeding^37,41,46^. Among the most important features of dihaploids is complete homozygosity. This allows the expression of rare recessive genes, the fixation of certain characters and properties, and their efficient transmission to the progeny^46^. The reduced DNA sequence complexity of haploids and homozygous dihaploids may also facilitate genome sequencing and assemblage^37,47,48^. The current draft genome sequence of *Ginkgo*, for example, was obtained from female megagametophyte tissue^49^. That tissue is certainly less handy for routine DNA extraction than leaves.

### Polyploids

Spontaneous triploid or tetraploid plants are found from time to time in many regularly diploid species and are also rarely reported among some gymnosperms^17,18,50^. *Ginkgo* may be now included to these rare examples, suggesting that spontaneous polyploidy is possible in plants irrespective of their eventual genome conservatism over geological timescales. Moreover, the number of reported polyploid *Ginkgo* plants indicates spontaneous polyploidy is certainly not extremely rare. The long polyploidy-free evolutionary history of *Ginkgo* therefore must be due to limited survival and post-emergence counter-selection of polyploid plants, such as, for example, due to decreased belowground or aboveground competitive performance, stress tolerance, lack of mating partners, or lowered fertility.

Spontaneous triploids originate mostly from fertilization by unreduced gametes or from inter-ploidy crosses^51^, the latter option being impossible at the moment due to the immaturity of all documented *Ginkgo* polyploids (see also below). Though triploids seem to appear in lower frequency in our survey compared to haploids or tetraploids, this is perhaps only due to a selection bias in the nurseries. Triploids are likely indistinguishable from diploid seedlings and saplings, so they remain mostly neglected during selection of unusual *Ginkgo* plants worthy of further cultivation and propagation (Figs. 2 and 4).

The spontaneous *Ginkgo* tetraploids cannot perhaps originate in any other way than as a somatic mutation (polyploidization) at some developmental stage. For tetraploids grown up from seedlings, this polyploidization must have happened during embryo formation or early during seedling emergence. In cultivars Pendula Gruga or Jagged Jade, both known to have originated as a branch sport (spontaneous bud mutation) on a likely diploid maternal tree (Table 1), polyploidy has happened perhaps analogously to the dihaploidy events observed in *Ginkgo* haploids. Polyploid gymnosperms have also been produced artificially by colchicine treatment in several species^18,52^; however, we are not aware of any such successful attempts in *Ginkgo*.

The homogeneity of genome size in haploids and triploids indicates these are pure examples of whole-genome mutations. The variation in genome size found in tetraploids, however, indicates that polyploidy must be coupled here with aneuploidy or other kinds of DNA eliminations resulting in slight genome size reduction in some tetraploid cultivars compared to the ideally doubled genome size.

Spontaneous or artificial triploid or tetraploid gymnosperms are usually slow growing, sterile, and short lived^17,18^. There exist some older gymnosperm polyploids in *Pinus*, *Larix*, and *Picea* which have grown for many years and have eventually reached maturity^52,53^, but their slow growth and high sterility disfavored them from further breeding programs. Polyploidy as a whole is therefore considered as an unpromising path for gymnosperm tree breeding^18,52,54^. In contrast to this opinion, many *Ginkgo* triploids and tetraploids grow quite well (Fig. 4, Table 1). Knápek’s 15-year-old triploid, for example, tolerates yearly pruning to reduce its height to a “garden acceptable” 3.5 m, and Ožana´s 3-year-old, 140 cm high triploid shows growth similar to its same-age diploid siblings cultivated under the same conditions in the nursery (Fig. 4). The ~22-year-old mother plant of cv. Freak reaches only about 3.5 m. However, it is cultivated rather as a bonsai plant in a 300 l pot (Fig. 4), which substantially restricts its natural growth. The other seed-borne tetraploids in Josef Hahnl nursery grow as well as the diploids (all grafted on diploid rootstock) and the situation is similar with the Masaryk University tetraploid (ref. ^26^: Fig. 1a). Their vital growth and relatively large leaves may predestine some of these polyploids for commercial planting and leaf production for the pharmacological industry, especially if the polyploidy has any advantageous effect on the composition of pharmacologically important compounds. Conversely, synthetic preparation of *Ginkgo* polyploids may prove to be an effective way of breeding high leaf productive *Ginkgo* cultivars. These eventual breeding attempts may be highly accelerated if some of the existing triploid trees prove to be fertile. Triploids may produce gametes with variable ploidy levels^51^ (haploid, diploid, or unreduced triploid) and mating with such a triploid individual may therefore provide a large spectrum of polyploid plants for effective selection of desired properties.

Compared with the tetraploids originating from seeds, those originating from buds (Pendula Gruga, Jagged Jade) grow more slowly, which is consistent with observations from other polyploid tree gymnosperms (conifers). In addition to slow growth, the common characteristic of these *Ginkgo* plants and other conifer tetraploids is a tendency to pendulous or weeping growth, as documented, for example, in the old tetraploid tree of *Larix decidua* (ref. ^53^: Plate 1) or in tetraploid *Pinus sylvestris* (ref. ^52^: Fig. 1). These growth characteristics predestine these bud-originated *Ginkgo* tetraploids to be grown rather as curiosities in parks and personal gardens. However, the evolutionary potential of such plants as well as any other *Ginkgo* tetraploids should not be underestimated^17,18^.

The rare notes about spontaneous formations of female, fruit-bearing branches on regularly male (homogametic gender) trees^55^, for example, indicate that any *Ginkgo* individual bears genes for both sexes, irrespective of sexual chromosome dimorphism. As in other dioecious plants, the sex determination in *Ginkgo* therefore must depend only on regulation of gene expression^56–58^. Polyploidy may easily break down any gene regulation^59,60^, and there are several examples of dioecious diploids forming hermaphrodite individuals or even separate hermaphrodite species following induced or natural polyploidy^61–64^. We are thus looking forward to the first observation of the sexes of the *Ginkgo* tetraploids in our survey, which are all still quite young and immature. If one were to be a hermaphrodite able to produce viable seeds, it could be even considered to be a new *Ginkgo* species.

### Cell and stomatal size

Genome size (and thus also polyploidy) determines minimum cell size. Therefore, genome size and ploidy usually correlate with the size of specific cells where smaller size provides some functional advantage^65^, such as the guard cells of stomata in plants^29,31,32,66,67^. The correlations with genome size and ploidy may also appear to a lesser extent with the size of whole organs, especially when formed with relatively constant cell numbers^66,68–70^. *Ginkgo* is no exception in this respect, and ploidy level correlates in *Ginkgo* positively with leaf and stomatal size. Leaves of *Ginkgo* haploids seem always smaller compared to leaves of any other *Ginkgo*, while those of triploids and tetraploids are regularly among the largest observed in the surveyed *Ginkgo* plants (Figs. 2 and 5). The leaves of *Ginkgo* tetraploids also seem thicker, as sometimes observed in other polyploid plants^66,68^, and somewhat crispy. Such characteristics are perhaps all a consequence of increased cell size inside the leaves. Even though leaf size may vary with branch or plant age, with leaf position on the tree, and with cultivation conditions, these leaf characteristics can serve as a first-step identification of plants with unusual ploidies or polyploidized buds and branches.

Like the size of leaves, guard cells of stomata are remarkably small in haploids and their size generally increases with increasing ploidy level. Because stomatal size is much conservative and less dependent on cultivation conditions^71^ compared to leaf size, its measurement can provide an even better alternative for ploidy identification in *Ginkgo*, at least for haploids and tetraploids. However, one must be careful to account for the partly sunken placement of *Ginkgo* stomata and to precisely estimate the guard cell edges in observed epidermal peels. Despite this methodical difficulty, we hope measurements of guard cells combined with leaf morphology can facilitate the discovery of other haploid and polyploid *Ginkgo* plants that may still remain hidden in various other *Ginkgo* nurseries and collections. Based on images on the Internet, for example, good candidates for further tetraploids seem to be cv. Crispin´s Jaded Jester, cv. Medusa and for haploids cv. Gokusho-ba.

Stomatal sizes of *Ginkgo *triploids and tetraploids are much larger than fast in any fossil Ginkgoales (ref. ^71^: Table S1; ref. ^72^: Table S1; ref. ^26^). The exception represent two samples of Jurrasic *Sphenobaiera*, whose pore size (~52.7 µm)^72^ is comparable to the pore length found in *Ginkgo* triploids and tetraploids (cf. Fig. S4). Although this finding suggests existence of polyploidy deep in the Ginkgoales history, the molecular evidence yet shows this was likely not the case of the extant *Ginkgo* lineage (accepting the critics^22^ of the previous evidence in the *Ginkgo* genomic data^21^). The reported polyploidy in *Ginkgo* therefore most probably represent an absolute evolutionary novelty in *Ginkgo*. The availability of multiple polyploid *Ginkgo* trees increases the chance some will be fertile and able to be further bred. If so, we may seriously start to think about breeding a new polyploid species of *Ginkgo* and to substantially accelerate the modern diversification of this fossil plant lineage.

## Electronic supplementary material

Tables S1-S5

Figures S1-S6
