# Supplementary material for: Multiple haploids, triploids, and tetraploids found in modern-day “living fossil” Ginkgo biloba
Source: Hortic Res. 2018 Oct 1;5:55. doi: 10.1038/s41438-018-0055-9 (PMC6165845; doi:10.1038/s41438-018-0055-9)
Supplement: Supplementary file 1 — Tables S1-S5 [file 41438_2018_55_MOESM1_ESM.pdf]

Table S1. Results of ploidy measurements in anonymous *Ginkgo* plants

Collection/collector	Plant desription	Ploidy	Number of screened plants
Iveta Hodová	two siblings of the Masaryk University tetraploid grown in personal garden in Ostrava	2x	2
Marie Kordíková	1 small >10 years old tree from personal garden	2x	1
Bohumír Pitthart	1 >80 years old tree from Opatovice nad Labem	2x	1
Martin Plhák	5 diploid plants grown up from seeds from female plants grown in Olomouc	2x	5
Kristýna Veselá	1 small >10 years old tree from personal garden in Čebín	2x	1
Ondřej Knápek	1 plant grown at personal garden in Bernartice nad Odrou, bought as a seedling in Tropik Hukvaldy garden shop (CR)	3x	1
Stanislava Milotová	1 >55 years old tree cultivated in personal garden in Poděbrady	2x	1
Pavla Kopřivová	1 small tree cultivated in personal garden in Štěpánovice nad Svratkou	2x	1
Alexandra Hejduková	1 plant from arboretum Makču Pikču in Paseka	2x	1
Petr Krčál	1 >20 years old bonsai plant bought in Botanical Garden in Praha	2x	1
Mr. Vojík	a tree sample form Praha	2x	1
Jiří Řehořka	15 years-old tree of ?cv. Mariken grown in personal garden in Praha	2x	1
Jiří Řehořka	25 years-old tree grown in personal garden in Humpolec	2x	1
Pavel Veselý	1 tree from Brno, Životského street	2x	1
Jana Blažíčková	73 two-years old samplings grown up from seeds of one female plant in Hybešova street in Brno	2x	73
Jana Blažíčková	1 two-years old samplings grown up from seeds of one female plant in Hybešova street in Brno	4x	1
Jana Blažíčková	4 eight or nine-years-old saplings grown up from seeds sampled in Vienna (Austria), Hybešova street in Brno, or the Botanical Garden of Mas	2x	4
Jana Blažíčková	1 older un-named plant bought in Hornbach garden shop in Brno	2x	1
Jana Blažíčková	54 ± five-years old samplings grown up from seeds sampled in Brno	2x	54
Jana Blažíčková	10 saplings grown up from seeds sampled in Vienna (Austria), Hybešova street in Brno, or the Botanical Garden of Masaryk University in Brn	2x	10
Vladimír Ožana	110 two to three years-old samplings grown up from seeds of mother plants from Olomouc	2x	110
Vladimír Ožana	1 three years-old samplings grown up from seeds of a mother plant from Olomouc	3x	1
Vladimír Ožana	10 about 20-years old trees grown up from bought seeds	2x	10
Vladimír Ožana	6 un-named trees of various age and origin grown in personal garden	2x	6
Vladimír Ožana	1 small leaved sedling cultivated from seeds from mother plant in Prostějov	2x	1
Petr Šmarda	1 6-years old sapling originated from seeds of female trees in the Botanical Barden of the Masaryk University in Brno, not measured by Šmar	2x	1
Petr Šmarda	1 unnamed plant from Bauhaus shop in Brno-Ivanovice	2x	1
Petr Šmarda	1 large tree growing at Brno-Poříčí (at the entrance to Czechglobe institute)	2x	1
Petr Šmarda	2 un-named upright growing trees in Otnice	2x	2
Herrenkampen Garden	4 saplings cultivated from seeds from China	2x	4
Herrenkampen Garden	2 saplings form seeds of unknown origin	2x	2
BG Budapest	5 old trees grown in the garden	2x	5
Jan Sláma	3 small tees with panashed leaves	2x	3
Jan Sláma	1 tree cultivated in the home town	2x	1
Jan Sláma	1 unnamed sample from USA	2x	1
Jan Sláma	2 rootstock plants	2x	2
BG Nitra	3 unnamed young trees	2x	2
BG Nitra	rootstock plant of cv. Tubifolia	2x	1
Eva Hettenbergerová	1 tree from bank of Balaton lake in Tihany (Hungary)	2x	1
Ondřej Knápek	1 tree form Práce village near Šlapanice u Brna	2x	1
BG Budapest	3 young, unnamed, grafted variegated saplings	2x	3
Tordas collection	1 rootstock plant cultivated from seeds of old trees grown in BG Budapest	3x	1
Katarína Ražná & Pavel Hrubík	Czech Republic, Prague Botanical Garden of the Charles University, tree number 17; age 60 years; height 4.5 m (formerly planted as a bonsai	2x	1
Katarína Ražná & Pavel Hrubík	Prague, Botanic garden of The Charles University, Czech Republic, tree number 15; age about 40 years; trunk diameter/circumference at bre	2x	1
Katarína Ražná & Pavel Hrubík	Krakow, Botanic Garden, Poland, tree number 5, female	2x	1
Katarína Ražná & Pavel Hrubík	Krakow, Botanic Garden, Poland, tree number 4, female; one of the oldest tree in Poland, tree trunks "czi-czi"	2x	1
Claudine Decuyper	oldest European tree of cv. Pendula grown in castle park in Enghien, Belgium	2x	1
Own sowings	1533 seedlings grown up from 3570 seeds of three possible maternal trees of the known tetraploid sapling found in the Botanical Garden of	2x	1533

Table S2. Locality and basic characteristics of screened Slovakian *Ginkgo* trees (all diploid)

Sample code	Locality	Sex	Age (years)	Height (m)	Trunk diameter at breast (130 cm) height (cm)	Trunk cimcurference at breast (130 cm) height
R1	Senné,district Veľký Krtíš, private garden; tree grown from seeds	juvenile	55	12	13.5	42
R2	Súdovce, district Krupina	male	150-200	14	96.5	302
R4	Trnava, park of A. Bernolák, at the memorial of A. Bernolák	male	72	15	42	131
R7	Banská Štiavnica, Botanic Garden	female	90	20	54	170
R10	Topoľčianky, Park of State Forest, at the chateau	female	114	20	82	256
R11	Topoľčianky, Park of State Forest, meadow at the chateau	female	132	18	70	220
R14	Nová Ves nad Žitavou, historic park at the chateau	female	116	16	85	266
R15	Nová Ves nad Žitavou, historic park at the chateau, near the gree	female	140	17	68	213
R16	Nová Ves nad Žitavou, historic park at the chateau, at the pond	female	87	20	53	165
R19	Trenčín, area for construction at the city park	male	185	16	103	325
R20	Trenčín, area for construction at the city park	female	147	16	65	204
R21	Záblatie, park at the restaurant	female	178	15	58	180
R22	Adamovské Kochanovce, new plantings	juvenile	13	4	10	25
R23	Kočovce, historic park at the chateau	female	104	25	73	230
R24	Rakovice, park in the Middle School of Gardening	female	120	20	75	237
R25	Piešťany, areal of the Middle School of Gardening	male	17	10	10	30
R26	Piešťany, areal of the Middle School of Gardening	female	44	15	28	89
R28	Piešťany, Spa park, left, first out of the way	female	142	20	89	280
R29	Piešťany, Spa park, right, second out of the way	female	120	25	60	188
R30	Oponice, historical park; crown width 8 m	male	94	137	59	185
R31	Kovarce, historical park	male	85	19	52	163
R32	Prievidza, city park	female	65	17	39	122
R34	Bojnice, park at the chateau, at the lake in the park, tree number	male	110	19	68	214
R35	Bojnice, park at the chateau, at the lake in the park, tree number	male	140	20	92	285
R36	Janova Ves, historic park at the chateau	male	197	16	118	370
R37	Bánovce nad Bebravou, city park	female	51	18	27	85
R38	Bánovce nad Bebravou, city park	male	112	20	60	187
R39	Šurianky, Park of the District Office	male	44	16	28	87
R40	Šurianky, Park of the District Office	male	60	14	32	99
R48+R158	Lučenec, Ipeľské tehelne, cv. Ohatsuki	female	135	22	85	267
R99	Spišská Belá, park at the museum	male	100	16	63	198
R131	Veľký Blh, historic park at the chateau	female	94	14	61	198
R134	Banská Štiavnica, Botanic Garden	female	?	22	58	183
R135	Nová Ves nad Žitavou, historic park at the chateau	female	140	18	88	277
R138	Arborétum Mlyňany, tree number 15	juvenile	26	14	17	53
R139	Arborétum Mlyňany, tree number 9	female	36	12	22	70
R140	Nová Ves nad Žitavou, historic park at the chateau	female	115	16	73	228
R143	Janova Ves, historic park at the chateau	male	197	22	111	373
R144	Sereď, private garden	juvenile	30	12	19	59
R145+R148	Hájna Nová Ves, park at the chateau	female	242	22	152	478
R151	Topoľčianky, Park of State Forest, at the chateau	female	132	18	83	261
R153	Nitra, Levická street, at the guesthouse	juvenile	12	6	5	15
R154	Nitra, City Park	female	60	16	37	116
R155	Nitra, City Park	male	60	12	23	70
R159	Nitra, Botanic Garden of Slovak University of Agriculture in Nitra,	juvenile	33	4	?	?
R160	Voderady, historic park at the chateau	male	148	18	93	292
R161	Hokovce, historic park at the chateau	female	84	16	53	166
R165	Banská Štiavnica, Botanic Garden, in the middle	male	112	18	70	191
R168	Horenická Hôrka -Medné, historic park at the chateau	female	158	25	110	345
R169	Klobušice, historic Park at the chateau	female	84	13	53	165
R170	Hokovce, historic Park at the chateau	male	112	25	70	220
R171	Hokovce, historic Park, tree number 2	juvenile	18	8	11	35
R172	Palárikovo, tree number 1, historic park at the chateau	male	160	20	101	316
R174	Palárikovo, tree number 4, historic park at the chateau	male	125	20	79	247
R175	Palárikovo, tree number 2, historic park at the chateau	female	31	10	20	62
R176	Palárikovo, tree number 3, historic park at the chateau	female	31	12	19	60
R179	Brezová pod Bradlom, in the areal of Elementary school	male	65	16	54	168
R180	Tomášikovo, historic park at the chateau	female	162	25	100	315
R181	Trnava, park of A. Bernolák, at the memorial of A. Bernolák	male	70	16	43	136
R182	Senica, Hurbanova street	juvenile	16	6	10	12
R183	Piešťany, Winterova street	male	142	25	89	280
R184	Šamorín, Slnečná street, private garden	male	42	14	54	169
R187	Brusno, at wooden pavilions of the Spa areal	juvenile	12	3.5	3	9
R191	Košice, Park of J.A. Komenský	male	158	20	100	312
R195	Budimír, historic park	male	79	22	49	155
R197	Trenčín	female	147	16	93	290
R198	Nová Dubnica, tree number 1	juvenile	19	3.5	7	21
R201	Klobušice, historic park	male	91	22	57	179
R203	Nová Dubnica, tree number 4	juvenile	19	4.5	11	33
R204	Trenčín	male	185	16	116	365

R206	Slovesnké Pravno, Elementary school	juvenile	12	1	1	3
R207	Rajecké Teplice, Spa Park	juvenile	10	3	5	15
R208	Martin, at the hotel Turiec	male	93	18	57	180
R217	Nitra, Kupecká street; alley tree	juvenile	12	5	12	30
R218	Nitra, Kupecká street; alley tree	juvenile	12	5	12	30
R209 (189)	Abramová	female	121	14	76	238
R210(188)	Mošovce, historic park at the chateau	male	65	12	40	125
R22/A	Adamovské Kochanovce, new plantings	juvenile	17	13	10	30
RBZ 2	Nitra, Botanic Garden of Slovak University of Agriculture in Nitra	juvenile	16	13	15	32

Table S3. Results of ploidy screening of various *Ginkgo* cultivars

Cultivar name	Screened collection(s)*	Observed ploidy(ies)	Number of screened plants
Adam	A	4x	2
Allgold	H	2x	1
Anny's Dwarf	HWBL	1x>2x (HWB), 2x (L)	5
Anny's Zebra	HL	2x	2
Aureovariegata	H	2x	1
Autumn Gold	HT	2x	2
Babos	B	2x	1
Baldii	HLW	1x (HL), 1x>2x (W)	4
Barabit´s Gift	H	2x	1
Barabit´s Pilar (Pillar)	BT	2x	2
Barabit's nana =?Barabitsii	H	2x	1
Barabits fastigiata	HLW	1x (HL), 1x>2x (W)	3
Barabits Sztráda = Globus	T	2x	1
Barabitsii	W	2x	1
Beijing Gold	HUB	2x	3
Ben	A	2x	1
Bergen Op Zoom	H	2x	2
Bernheim Arboretum	B	2x	1
Bernheim Broom	WB	2x	2
Berthold Leerderlz	H	2x	1
Blagon	B	2x	1
Boleslaw Chrobry	W	2x	1
Brooks	H	2x	1
Broom With Tubes	H	2x	1
Buddy	H	2x	1
Bullwinkle	W	4x	1
Busse	H	2x	1
BZN	W	2x	1
California Sunset	HB	2x	2
Canopy	H	2x	1
Celeia	H	2x	2
Clica (=Folker´s Selection)	HWLA	1x	5
Compacta	W	2x	1
Curly Leafs	A	4x	2
D. Js Bow Tie	WB	2x	1
David	H	2x	1
Denise	H	2x	1
Diamant	B	2x	1
Dila	H	2x	1
Dist	H	2x	1
Domhoff-Freysing	W	2x	1
Dr. Faustus	A	2x	1
Dr. Gerd Krüssmann	H	2x	1

Eiffel	B	2x	2
Eladua	B	2x	1
Elmwood	HB	2x	3
Elsie	W	2x	1
Everton	B	2x	1
Everton Broom	W	2x	1
Fairmount	HT	2x	3
Fastigiata (=Sentry)	HNLTWC	1x (N), 1x>2x (W), 2x (CNTL)	6
Fastigiata Blagon	HW	2x	2
Fastigiata Tilburg	A	2x	1
Fillips perfekt spire	W	2x	1
Fillips weeping	H	2x	1
Finger (grün)	WA	2x	2
Finger variegated	HB	2x	3
Freak	HBA	4x	3
Giant Spreader	B	2x	1
Girad´s Spreader	H	2x	2
Glen Dwarf	B	2x	1
Globosa	HN	2x	3
Globus	H	2x	2
Gnome	H	2x	1
Golden Colonade	H	2x	1
Golden girl	H	2x	1
Golden globe	WBL	2x	2
Goldstein Globe	H	2x	1
Green Pagoda	H	2x	1
Gretchen	A	2x	1
Hayanari	HB	2x	2
HB Leiden	HT	2x	2
HB-Trompenburg	H	2x	2
Heterophylla	H	2x	2
Hettych	BT	2x	3
Hop Sing	A	2x	1
Horizontalis (Dieck´s)	H	2x	1
Horizontalis (Hahnl´s)	A	4x	2
Horizontalis nana	H	2x	2
Hoss	A	4x	2
Hunnewell broom	WL	2x	2
Chase Manhattan	HW	1x>2x	2
Chotek	H	2x	1
Chris Dwarf	WOLB	1x (BOL), 1x>2x (W)	4
Itebych	B	2x	1
Jade Butterfly	HB	2x	2
Jagged Jade	W	4x	1
Jehosaphat /spring grove	WB	2x	2
Katlan	HW	2x	2
King of Basting	B	2x	2

King of Dongting	HB	2x	2
Kitsi	HBT	2x	4
Kohout Compact	L	2x	1
Kohout’s pendula WB	H	2x	1
Kopista	W	2x	1
Korinek	HST	2x	4
Kristina	HB	2x	2
Kroměříž	B	2x	1
Lake Butterflies	H	2x	1
Lakeview	HBT	2x	3
Landliebe	WL	2x	2
Late fall gold	H	2x	1
Lehda	W	2x	1
Lída	S	2x	1
Linea	T	2x	2
Little Joe	HWA	1x (H), 1x>2x (W,A)	5
Long March	W	2x	1
Louis	H	2x	1
Löver	W	2x	1
Magnifica	HB	2x	2
Magyar	HBT	2x	3
Majestic butterfly	W	2x	1
Majestic Weeper	H	2x	1
Maribo	B	2x	1
Mariken	HBSLO	2x	6
Maro	W	2x	1
Marona	H	2x	1
Mayfield	HT	2x	2
Maytown	H	2x	1
Menhir	BHLA	1x (BL), 1x>2x (HA)	5
Mephisto	HBA	2x	4
Microsperma	HB	2x	2
Montezuma	H	2x	1
Munchkin	BHW	1x (BH), 1x>2x (W)	3
Mutant weeper	HBW	2x	3
Nelleke	HB	2x	2
Nory´s	B	1x	2
Obelisk	HBL	1x	5
Ohazuki / epiphylla	HTW	2x	4
Originalis 1983	W	2x	1
Orlóci klón	T	2x	1
Oszlopos Tekeres	HT	2x	2
Pagoda	L	2x	1
Palo Alto	HT	2x	2
Paul Vaught	B	2x	1
Pendula	HUNL	2x	5
Pendula Gruga	HBN	4x	5

Pendula variegata	HB	2x	2
Peve greenleave	W	2x	1
Peve Lobo	HB	2x	2
Peve Maribo	W	2x	1
Peve twisted (=Kronkel)	WB	2x	2
Pine Glen Dwarf	H	2x	1
Pine Golden Dwarf	B	2x	1
PNI	B	2x	1
Pragense	HBSL	2x	3
Princeton Sentry (=PNI2720)	HB	2x	2
Prostrata	S	2x	2
Pyramis	HT	2x	2
Robie´s twist	HB	2x	2
Rocky	H	1x	1
Ronald’s Snowfall	H	2x	1
Roos More	HB	2x	3
Roswitha	H	2x	2
Salve	W	2x	1
Samurai	W	2x	1
San Jose	W	2x	1
San Jose Gold	HBT	2x	3
Sandrandiga	A	2x	1
Saratoga	HBTC	2x	6
Saratoga Broom	H	2x	1
Sarj	B	2x	1
Saskatchewan	H	2x	1
Selection	H	2x	2
Selection Select	HB	2x	2
Sendei Chi Chi	A	2x	1
Shangri-La	H	2x	1
Schloss Dyke	H	2x	1
Schönbrunn	H	2x	1
Simon	HB	2x	2
Sinclair	H	2x	1
Saint Cloud (Skeleton)	B	2x	1
Sky Tower / Sky Walker	W	2x	1
Sortaja	H	2x	1
Sotayu	W	2x	1
Sport horizontalis	WB	2x	2
Sport Tit	H	2x	1
Spring Grove	H	2x	1
Spring grove sport	WB	2x	2
Stanley’s Dwarf	W	2x	1
Summer Rainbow	H	2x	1
Sunstream	H	2x	1
Survivor	HL	2x	2
Thelma	HB	2x	3

Tit / chi-chi icho	HB	2x	2
TITZ	S	2x	1
Todd’s WB	HW	2x	2
Törpe Enlopes	H	2x	2
Tremonia	HT	2x	4
Troll	HBSL	2x	3
Tubifolia	HSNLT	2x	8
Umbrella (=Santa Crux)	HLT	2x	4
Vanilla swirl	H	2x	1
Vanisgate	H	2x	1
Variegata	HL	2x	1
W.B. - Witches Broom	W	2x	1
Weeping wonder	S	2x	1
Wiener Waltzer	BHT	2x	3
Windover gold	HB	2x	2
Yatsubusa	HB	2x	2
Yellow Dragon	HL	2x	2
Yellow mellow	H	2x	1

* Abbreviations of screened collections:

A – Josef Hahnl tree nursery in Chorherrn , Austria

B – Botanical Garden of Eötvös University in Budapest, Hungary (http://www.fuveszkert.com)

C – Hornbach shop in Brno, Czech Republic
H – Herrenkamper Gärten, Germany (http://pflanzenraritaeten.com)
L – Ovocné a okrasné školky Litomyšl grden shop, Czech Republic
N – Botanical Garden of the Slovak University of Agriculture in Nitra, Slovakia
O – Vladimír Ožana tree nursery in Bílov, Czech Republic
S – Jan Sláma dwarf tree nursery , Czech Republic (http://wbgarden.com/nove/ei2.htm)
T – Collection of the Hungarian National Food Chain Safety Office in Tordas, Hungary
U – Bauhaus shop in Brno-Ivanovice, Czech Republic
W – Private Ginkgo collection of Dr. Manfred Bindewald in Erlenbach am Main, Germany (https://m.bindewald.net/Ginkgoarboretum.php)

Table S4. Relative genome sizes of *Ginkgo* plants and cultivars with unusual ploidies measured using flow cytometry and DAPI dye

Sample	Collection*	Internal standard	Ploidy level	Relative genome size (Sample/standard ratio)	CV of standard peak (%)	CV of sample peak (%)
Nory's	B	***Pisum sativum***	**1x**	1.377	1.47	1.68
Anny's Dwarf	H	***Pisum sativum***	**1x**	1.378	1.44	1.51
Little Joe	H	***Pisum sativum***	**1x**	1.379	2.01	1.66
Rocky	H	***Pisum sativum***	**1x**	1.384	1.64	1.58
Menhir	H	***Pisum sativum***	**1x**	1.386	1.87	1.80
Barabit's Fastigiata	W	***Pisum sativum***	**1x**	1.386	1.53	1.51
Chris's Dwarf	H	***Pisum sativum***	**1x**	1.387	1.35	1.50
Fastigiata	W	***Pisum sativum***	**1x**	1.388	1.74	1.63
Clica	W	***Pisum sativum***	**1x**	1.389	1.48	1.44
Obelisk (plant 2)	H	***Pisum sativum***	**1x**	1.389	2.20	1.39
Baldi	W	***Pisum sativum***	**1x**	1.390	1.27	1.37
Folker Select (=Clica)	W	***Pisum sativum***	**1x**	1.391	1.69	1.48
Obelisk (plant 1)	H	***Pisum sativum***	**1x**	1.391	1.47	1.32
Chase Mannhatan	H	***Pisum sativum***	**1x**	1.396	1.20	1.12
Munchkin	W	***Pisum sativum***	**1x**	1.403	1.71	1.69
Ožana´s triploid	O	***Vicia faba***	**3x**	1.310	1.73	1.42
Orlóci triploid	T	***Vicia faba***	**3x**	1.311	1.52	1.60
Knápek´s triploid	Personal collec	***Vicia faba***	**3x**	1.316	1.86	1.37
Pendula Gruga	N	***Vicia faba***	**4x**	1.729	1.39	1.84
Pendula Gruga (plant 1)	H	***Vicia faba***	**4x**	1.735	1.43	1.69
Pendula Gruga (plant 2)	H	***Vicia faba***	**4x**	1.743	1.11	1.27
Masaryk University tetraploid	BG Brno	***Vicia faba***	**4x**	1.745	1.93	2.16
Bullwinkle	W	***Vicia faba***	**4x**	1.746	1.84	1.97
Blažíčková´s tetraploid	Personal collec	***Vicia faba***	**4x**	1.763	1.17	1.27
Freak (plant 1)	H	***Vicia faba***	**4x**	1.798	1.62	1.81
Freak (plant 2)	H	***Vicia faba***	**4x**	1.799	1.52	1.54
Freak	H	***Vicia faba***	**4x**	1.806	2.03	1.35
Jagged Jade	W	***Vicia faba***	**4x**	1.813	1.93	1.57

* The coding follow Table S3

Table S5. Stomatal guard cell lengths of selected *Ginkgo* plants/cultivars

			Guard cell (stomatal) length (µm)						Stomatal width (µm)					
Sample Nr.	Cultivar/plant	Ploidy level	Mean	Minimum	25% quantile	Median	75% quantile	Maximum	Mean	Minimum	25% quantile	Median	75% quantile	Maximum
1	Folkers Select (W)	1x	38.44	31.76	36.40	38.81	40.22	44.27	30.63	25.45	28.79	30.37	31.94	39.43
2	Rocky (H)	1x	39.56	29.80	34.87	40.20	42.97	60.67	31.01	24.40	29.11	30.77	33.35	37.61
3	Baldi (sample 1, L)	1x	39.60	31.48	37.50	39.11	41.53	46.49	31.13	22.51	28.41	30.75	32.70	44.04
4	Anny´s dwarf (L)	1x	39.73	32.89	37.56	40.32	42.16	47.71	30.58	25.27	28.28	30.59	32.16	37.66
5	Chase Manhattan (H)	1x	39.80	32.06	36.58	38.99	42.60	52.23	32.64	27.74	30.59	32.41	34.54	39.52
6	Nory´s (B)	1x	39.80	30.86	36.66	40.40	43.93	47.31	31.70	25.92	29.73	31.36	33.64	39.78
7	Fastigiata (N)	1x	40.77	32.23	37.94	40.58	44.09	47.69	31.50	25.52	29.73	30.98	33.79	36.89
8	Baldi (sample 2, L)	1x	41.54	31.55	38.38	40.87	45.13	52.94	32.47	27.39	30.57	31.89	34.34	38.42
9	Little Joe (H)	1x	41.67	31.02	38.22	42.26	45.35	50.49	32.05	26.45	30.49	31.80	34.13	38.17
10	Barabits fastigiata (W)	1x	41.97	31.41	39.73	41.88	44.31	51.98	30.56	22.58	27.99	31.01	32.71	39.66
11	Menhir (B)	1x	42.13	34.28	40.16	41.80	44.48	50.49	29.63	24.47	28.09	29.58	31.36	35.37
12	Munchkin (W)	1x	42.27	32.47	40.09	42.34	45.86	48.91	34.39	26.99	29.97	34.15	39.16	44.17
13	Obelisk (L)	1x	42.41	28.23	40.10	42.54	45.07	55.04	33.16	25.52	31.37	33.03	35.12	40.54
14	Chris dwarf (H)	1x	42.52	33.26	39.31	42.30	46.03	51.01	31.93	22.91	29.40	32.48	34.58	41.77
15	Clica (L)	1x	42.54	31.55	38.76	42.28	46.29	54.25	31.14	24.22	28.99	30.71	33.49	40.06
16	Chris dwarf (O)	1x	44.47	39.23	42.11	44.73	45.94	51.05	31.92	24.40	29.89	31.96	33.93	38.99
17	Maribo (B)	2x	45.90	34.16	42.15	45.61	49.79	55.91	38.19	30.36	35.99	38.27	39.68	51.08
18	Troll (B)	2x	46.52	35.96	43.91	46.46	49.52	55.88	33.68	29.72	31.91	33.47	35.20	38.76
19	Eladua (B)	2x	47.05	36.84	42.96	47.22	49.49	63.60	37.11	31.01	35.01	36.86	38.99	45.44
20	Everton (B)	2x	47.05	37.98	44.26	47.02	49.86	54.99	37.21	31.17	34.51	37.31	39.69	44.85
21	Saratoga (T)	2x	47.51	36.16	44.27	47.42	50.60	59.71	36.64	29.16	35.05	36.00	38.64	43.75
22	Glen Dwarf (B)	2x	47.98	39.37	44.39	47.89	50.63	60.50	38.86	30.50	36.39	38.12	41.23	46.93
23	Wiener Walzer (T)	2x	48.11	36.20	43.76	48.75	51.29	59.82	39.52	32.46	37.04	39.53	42.12	48.07
24	old tree (B)	2x	49.00	41.50	45.63	47.99	51.02	63.24	40.03	33.98	37.81	39.08	42.03	47.65
25	Mariken (B)	2x	49.22	40.61	46.23	48.74	52.25	60.61	38.06	28.42	36.26	38.21	39.50	46.98
26	Troll (H)	2x	49.30	37.63	45.53	49.83	53.18	60.19	38.58	28.89	35.71	39.04	40.84	49.02
27	Palo Alto (T)	2x	49.44	36.29	45.34	49.57	53.66	62.47	39.79	27.88	37.87	39.67	42.48	47.06
28	Fastigiata (T)	2x	49.51	41.29	46.29	49.20	52.72	58.11	39.81	32.56	37.71	39.55	42.11	48.11
29	Tubifolia (N)	2x	49.75	41.41	46.52	49.74	52.26	58.86	37.81	31.24	36.07	37.82	39.01	46.82
30	King of Donting (B)	2x	51.01	40.52	47.73	50.08	53.05	62.22	40.56	34.56	38.39	40.71	42.53	48.54
31	Umbrella (H)	2x	51.20	42.56	48.59	51.17	53.54	57.94	39.83	33.49	37.78	39.35	41.31	48.30
32	Giant Spreader (B)	2x	51.72	39.14	48.32	52.44	54.56	62.29	40.32	31.12	38.78	40.19	41.99	47.53
33	3 years-old-sapling (O)	2x	52.21	43.01	48.79	51.20	55.71	66.38	42.17	34.60	38.92	40.95	45.37	52.80
34	Yellow dragon (B)	2x	53.97	41.19	51.61	54.11	57.58	62.73	40.06	34.15	38.10	40.37	41.73	48.65
35	Golden Globe (B)	2x	54.88	45.24	51.75	53.71	57.35	68.90	42.39	33.84	39.64	41.87	44.58	53.83
36	Kristina (H)	2x	55.64	45.54	52.71	55.94	59.28	67.45	36.23	30.27	35.00	36.35	37.78	40.64
37	Skeleton (B)	2x	55.69	39.67	51.93	56.40	59.01	65.06	44.23	37.55	41.67	44.03	46.24	52.53
38	Orlóci triploid (T)	3x	53.25	43.78	50.05	53.26	56.40	63.75	41.51	36.23	39.10	40.89	43.79	51.09
39	Ožana´s triploid (O)	3x	57.61	44.35	53.88	58.37	61.59	69.09	40.56	33.23	37.95	39.71	43.19	53.16
40	Knapek´s triploid	3x	64.87	53.48	60.14	65.70	69.29	78.24	45.89	39.33	42.57	45.36	47.68	63.60
41	Blazickova´s tetraploid	4x	61.42	51.82	57.51	61.86	64.57	73.46	42.71	33.21	38.40	42.74	45.32	57.15
42	Pendula Gruga (N)	4x	61.47	46.12	56.91	61.26	65.31	81.69	48.85	40.27	45.56	47.64	52.77	60.86
43	Adam (A)	4x	63.50	53.24	59.90	63.71	67.23	78.33	47.73	40.02	43.90	47.57	50.50	56.53
44	Freak (H)	4x	63.61	48.78	59.17	63.29	67.78	81.74	48.05	37.06	44.29	47.82	51.36	59.85
45	Hoss (A)	4x	65.88	48.54	60.13	67.14	70.56	85.09	49.87	39.00	47.16	50.20	52.12	58.52
46	Jagged Jade (W)	4x	66.27	43.05	61.67	65.31	71.51	83.14	47.11	35.82	43.40	46.05	49.72	73.07
47	Curlie Leafs (A)	4x	67.10	55.01	62.54	67.38	71.16	79.91	49.84	41.07	47.67	50.21	51.91	61.43
48	Horizontalis (A)	4x	67.89	54.97	65.74	67.46	70.70	87.69	48.99	41.25	46.00	48.73	52.22	57.86
49	Bullwinkle (W)	4x	67.95	48.30	62.29	67.97	72.43	87.08	51.17	40.80	48.70	50.64	54.04	59.81
50	Masaryk University tetraploid	4x	70.92	58.45	67.02	70.79	73.95	86.39	51.40	41.49	47.78	51.27	54.19	63.48
51	Pendula Gruga (B)	4x	71.58	55.67	68.38	71.70	74.54	85.88	49.67	43.03	47.22	49.20	52.17	57.74

	Stomatal area (μm^2; length * width)						Stomatal pore length (µm)					
Sample Nr.	Mean	Minimum	25% quantile	Median	75% quantile	Maximum	Mean	Minimum	25% quantil	Median	75% quantil	Maximum
1	1178	867	1081	1173	1275	1563	32.57	11.75	28.49	32.81	36.91	47.29
2	1235	727	1054	1220	1368	2153	38.98	28.72	36.26	38.21	42.27	49.34
3	1236	812	1074	1209	1361	1979	40.94	28.55	34.21	42.60	45.52	54.51
4	1217	919	1090	1204	1340	1680	31.79	22.75	28.62	30.82	34.32	47.69
5	1305	903	1153	1248	1444	1789	31.96	24.69	28.11	30.80	35.74	49.07
6	1268	800	1111	1277	1420	1758	36.21	28.93	34.29	35.96	38.56	45.78
7	1289	870	1144	1297	1448	1694	37.22	27.06	34.98	37.13	40.16	49.38
8	1351	1039	1179	1301	1468	1843	32.02	18.23	27.27	31.50	35.79	51.94
9	1338	934	1177	1358	1491	1722	35.85	18.99	33.40	35.84	39.31	49.18
10	1284	881	1113	1260	1429	1706	35.47	25.59	31.86	36.46	38.47	45.89
11	1248	892	1176	1243	1338	1568	39.57	28.71	36.81	39.34	42.03	50.31
12	1451	1127	1262	1404	1640	1925	34.63	22.78	30.63	34.92	38.71	42.71
13	1409	831	1226	1438	1538	1929	39.10	27.13	36.12	39.33	42.41	49.57
14	1370	762	1182	1399	1561	2046	38.37	27.65	35.62	38.73	41.49	50.23
15	1332	940	1139	1292	1516	2029	38.49	30.37	35.59	38.30	40.56	47.87
16	1421	1006	1292	1401	1524	1951	41.18	30.57	37.40	41.11	45.13	52.21
17	1761	1055	1596	1737	1942	2802	35.35	27.62	32.31	35.23	37.98	44.90
18	1567	1107	1462	1575	1671	2112	40.17	25.67	36.45	40.24	45.12	48.32
19	1753	1204	1521	1728	1904	2399	38.09	30.05	35.68	37.08	40.74	47.31
20	1760	1193	1549	1680	1929	2466	39.86	31.97	36.36	40.23	41.26	51.70
21	1745	1219	1597	1749	1875	2426	47.12	35.54	42.77	47.48	49.87	68.79
22	1875	1201	1644	1863	2030	2737	40.53	31.20	37.75	41.77	44.22	48.93
23	1911	1330	1617	1901	2206	2564	42.55	32.88	38.66	43.37	46.64	51.10
24	1966	1484	1792	1919	2114	2893	46.47	35.52	43.67	47.19	49.20	58.94
25	1877	1299	1708	1828	2093	2506	40.22	25.58	35.57	40.16	43.91	58.80
26	1912	1178	1644	1934	2177	2862	39.72	25.27	34.92	39.29	44.27	51.77
27	1977	1196	1741	1998	2258	2705	43.03	34.26	40.38	42.10	46.02	52.44
28	1978	1441	1791	1956	2141	2723	44.95	34.16	41.48	45.96	47.66	54.16
29	1883	1451	1713	1875	1997	2395	41.23	28.90	37.44	40.54	43.58	54.92
30	2073	1641	1875	2027	2191	2775	46.05	37.36	43.55	45.68	49.50	54.38
31	2037	1569	1942	2070	2119	2500	42.70	26.54	38.91	42.41	47.52	58.04
32	2087	1518	1905	2085	2236	2805	46.74	36.46	43.44	46.87	50.22	58.76
33	2210	1607	1900	2154	2443	3195	41.95	31.72	36.00	40.87	46.63	55.64
34	2165	1567	1999	2193	2324	2837	47.09	35.89	42.76	46.11	51.49	59.87
35	2326	1665	2124	2302	2552	3169	49.81	35.02	46.13	49.79	54.56	63.21
36	2016	1481	1873	2014	2177	2432	50.23	35.50	45.52	51.05	54.25	66.04
37	2466	1490	2339	2482	2662	3020	48.09	37.19	42.72	47.92	52.65	61.87
38	2217	1671	1987	2165	2390	3228	48.85	34.12	44.09	48.86	53.72	66.22
39	2335	1618	2124	2300	2568	3375	50.81	32.71	46.45	51.21	55.13	71.92
40	2984	2325	2666	2811	3236	4603	54.65	40.02	49.00	54.41	58.60	80.35
41	2633	1724	2337	2640	2899	3668	54.50	42.14	49.24	52.74	58.69	72.49
42	3020	1963	2575	3030	3427	4336	55.35	42.44	51.57	54.48	58.48	67.72
43	3031	2423	2725	3030	3306	3825	65.53	41.41	60.27	65.83	70.99	85.35
44	3070	1845	2718	3009	3370	4739	51.69	40.31	47.45	50.82	56.65	70.04
45	3290	1893	2886	3328	3609	4495	60.24	40.92	52.53	58.88	65.43	99.94
46	3116	1983	2788	3056	3352	4300	62.29	49.62	57.89	61.43	66.67	76.44
47	3345	2678	3091	3312	3587	4285	65.58	47.25	59.45	65.61	70.16	84.26
48	3325	2438	3014	3264	3610	4279	64.15	40.35	60.52	63.44	68.52	81.95
49	3487	2433	3157	3403	3774	5113	61.85	44.64	56.16	62.47	68.30	75.56
50	3636	2782	3444	3623	3864	4623	60.00	44.42	55.32	60.33	65.15	75.24
51	3551	2844	3329	3567	3736	4950	55.24	38.10	50.91	55.76	59.37	71.21

	Stomatal pore width (µm)						Stomatal pore area (μm^2; length * width)					
Sample Nr.	Mean	Minimum	25% quantil	Median	75% quantil	Maximum	Mean	Minimum	25% quantil	Median	75% quantil	Maximum
1	18.05	9.81	15.55	17.59	20.73	26.99	594	223	469	564	725	1067
2	19.68	14.05	17.91	19.87	20.72	26.27	768	474	679	771	838	1084
3	19.56	13.46	17.93	20.10	21.39	23.62	810	432	648	829	942	1166
4	18.06	13.59	16.31	18.30	19.64	23.49	576	328	510	582	624	888
5	18.39	13.89	16.59	18.23	19.75	23.43	589	393	500	570	674	1023
6	19.60	14.77	17.52	19.53	21.78	23.69	711	504	634	689	787	1039
7	20.06	13.53	18.77	19.83	21.45	25.61	747	484	664	733	826	1023
8	18.37	11.23	16.38	18.11	20.17	28.69	593	222	468	579	675	1285
9	18.58	13.44	16.66	18.41	20.18	26.57	668	352	569	656	758	1018
10	18.46	13.14	15.72	17.46	21.61	26.60	662	405	516	630	800	1184
11	18.34	12.69	17.09	18.26	19.58	23.06	728	450	650	726	800	1050
12	19.80	13.33	17.86	20.02	21.29	25.37	687	425	588	687	794	981
13	19.52	15.32	17.59	19.25	21.42	27.06	762	553	655	757	894	986
14	21.40	14.56	19.12	21.07	23.64	28.52	824	498	668	805	967	1217
15	17.92	13.33	15.72	17.28	20.15	24.23	692	469	615	662	818	988
16	22.09	15.12	18.87	21.84	24.68	31.97	911	534	773	892	1031	1438
17	23.70	19.73	21.78	22.62	25.24	30.50	837	579	735	804	886	1207
18	20.91	17.16	19.15	20.76	22.10	25.59	836	625	721	862	915	1159
19	24.04	19.13	21.86	24.00	25.86	29.67	918	713	807	880	992	1259
20	21.90	17.81	20.54	21.18	22.95	27.78	870	609	759	886	930	1146
21	26.78	19.67	25.58	26.99	28.68	30.87	1265	876	1084	1235	1414	2101
22	20.72	15.25	19.35	21.30	22.21	26.51	843	573	716	852	949	1140
23	25.52	19.97	23.45	25.14	27.47	30.24	1092	800	916	1029	1291	1416
24	25.24	19.75	22.96	24.54	27.51	31.77	1178	702	1054	1204	1291	1593
25	23.48	15.27	21.24	22.94	25.29	33.87	954	485	783	941	1069	1835
26	23.40	14.98	21.20	23.22	25.31	30.14	939	440	747	944	1083	1458
27	25.77	19.33	24.64	26.40	27.32	31.26	1113	710	1032	1130	1262	1450
28	26.83	23.76	24.94	27.05	27.63	33.51	1209	835	1061	1245	1331	1541
29	22.83	16.40	21.45	22.46	24.03	30.06	947	622	785	928	1037	1651
30	25.33	19.37	23.24	26.12	27.45	30.36	1173	724	1069	1144	1331	1603
31	25.72	17.60	23.13	25.32	28.34	36.99	1108	521	913	1106	1285	2024
32	25.04	14.98	22.66	24.41	28.40	30.41	1173	672	1059	1167	1288	1707
33	20.18	13.49	17.40	19.52	22.98	29.88	853	442	673	811	979	1367
34	22.83	17.94	20.06	23.42	24.84	31.33	1077	741	945	1038	1223	1545
35	26.25	21.29	23.88	26.35	28.15	32.05	1312	833	1138	1318	1503	1876
36	23.89	17.37	21.97	23.56	25.68	31.42	1205	679	1012	1173	1364	1915
37	26.44	21.74	23.74	26.92	28.29	31.33	1265	875	1094	1294	1404	1524
38	28.00	23.16	25.78	28.05	29.83	34.80	1370	875	1148	1310	1609	1947
39	25.58	17.93	24.09	25.76	26.95	32.71	1303	736	1117	1316	1453	1989
40	29.97	21.93	26.87	29.85	32.40	38.64	1641	1026	1384	1601	1860	2562
41	27.19	21.34	24.72	27.26	29.44	32.85	1484	981	1295	1439	1650	1975
42	30.89	25.78	29.16	31.21	32.19	35.70	1710	1157	1517	1659	1914	2248
43	30.00	20.71	27.36	29.57	31.86	39.04	1959	1322	1737	1890	2166	2781
44	26.50	17.47	23.54	26.54	28.99	36.43	1377	883	1133	1332	1552	2542
45	30.25	21.15	27.27	29.83	33.29	39.93	1828	1060	1525	1764	2050	3270
46	32.09	24.59	29.47	31.07	35.03	44.43	2009	1262	1735	1969	2262	2747
47	32.08	25.54	29.67	32.25	34.27	40.23	2099	1538	1882	2005	2316	2803
48	32.43	24.52	30.18	32.89	34.83	39.80	2086	1148	1849	2029	2367	2913
49	30.57	23.06	27.68	30.74	33.06	38.67	1896	1113	1645	1918	2110	2505
50	29.65	24.35	27.44	29.07	32.26	35.23	1780	1247	1558	1757	2020	2331
51	32.90	26.16	30.17	32.34	35.19	42.26	1822	1126	1616	1784	2040	2730

* – orgin of plant is indicated in brackets following the plant/cultivar name; the coding of collections follow Table S3.
