# Supplementary material for: Multiple haploids, triploids, and tetraploids found in modern-day “living fossil” Ginkgo biloba
Source: Hortic Res. 2018 Oct 1;5:55. doi: 10.1038/s41438-018-0055-9 (PMC6165845; doi:10.1038/s41438-018-0055-9)
Supplement: Supplementary file 2 — Figures S1-S6 [file 41438_2018_55_MOESM2_ESM.docx]

**Fig. S1.** Stomatal width of different *Ginkgo* samples (cultivars). Boxplots show: median (thick horizontal line), interquartile range (box), non-outlying values range (whiskers), and outliers (circles). The nine rightmost haploid samples show statistical similarity with the diploid dwarf *Ginkgo* sample 18, and sample 12 also with the five leftmost diploid samples (Tukey HSD test, p>0.05); triploids cannot be distinguished in general from diploids or tetraploids; tetraploid samples 41 and 46 show some statistical similarity to one or more diploid samples. Sample coding follows Table S5.

**Fig. S2.** Stomatal area (length*width) lengths of different *Ginkgo* samples (cultivars). Boxplots style follows Fig. S1, sample coding Table S5. The seven rightmost haploid samples show statistical similarity with the diploid dwarf *Ginkgo* sample 18, and the diploid sample 37 with the tetraploid sample 41 (Tukey HSD test, p>0.05); triploids cannot be distinguished in general from diploids or tetraploids.

**Fig. S3.** Comparison of measured stomatal and pore parameters (sample medians) using linear regression.

**Fig. S4.** Stomatal pore lengths of different *Ginkgo* samples (cultivars). Boxplots style follows Fig. S1, sample coding Table S5. All haploid samples show statistical similarity with one or more diploid samples (mostly dwarf *Ginkgo* cultivars), and the tetraploid samples 41, 42, 44, 51 also with one or more samples of diploids (Tukey HSD test, p>0.05); triploids cannot be distinguished in general from diploids or tetraploids.

**Fig. S5.** Stomatal pore width of different *Ginkgo* samples (cultivars). Boxplots style follows Fig. S1, sample coding Table S5. All haploid samples show statistical similarity with one or more diploid samples (mostly dwarf *Ginkgo* cultivars), and the tetraploid samples 41, 44, 50 also with one or more samples of diploids (Tukey HSD test, p>0.05); triploids cannot be distinguished in general from diploids or tetraploids.

**Fig. S6.** Stomatal pore area (length*width) of different *Ginkgo* samples (cultivars). Boxplots style follows Fig. S1, sample coding Table S5. The twelve rightmost haploid samples show statistical similarity with one or more diploid samples, and the tetraploid samples 41, 44 also with one or more samples of diploids (Tukey HSD test, p>0.05); triploids cannot be distinguished in general from diploids or tetraploids.
